# Book Notices

**Published:** 1895-09

**Authors:** 


### Book Notices.

P. Blakiston, Son & Co., of 1012 Walnut street, Philadelphia, announce that they have in preparation for early issue an authorized translation, by Dr. Albert B. Hale, of Chicago, of a Handbook of Diseases of the Eye, by Dr. A. Eugen Fick, of the University of Zurich. This is one of the most complete, thorough and compact of text-books. Among its other merits, it contains a number of very handsome colored illustrations, not of rare or unusual cases, but of practical matters that will greatly aid the student and be of much service to the practitioner. The retail price will be from $3.00 to $4.00.

